# Controlling the Solubility, Release Rate and Permeation of Riluzole with Cyclodextrins

**DOI:** 10.3390/pharmaceutics16060757

**Published:** 2024-06-03

**Authors:** Tatyana Volkova, Olga Simonova, German Perlovich

**Affiliations:** G.A. Krestov Institute of Solution Chemistry RAS, 153045 Ivanovo, Russia; vtv@isc-ras.ru (T.V.); ors@isc-ras.ru (O.S.)

**Keywords:** solubility, dissolution/release, permeation rate, artificial membranes

## Abstract

Riluzole (RLZ), a sodium channel-blocking benzothiazole anticonvulsant BCS class II drug, is very slightly soluble in aqueous medium. To improve aqueous solubility and modulate dissolution rate and membrane permeability, complex formation of RLZ with two cyclodextrin, α-cyclodextrin (α-CD) and sulfobutylether-β-cyclodextrin (SBE-β-CD), was studied. The stability constants demonstrated a greater affinity of SBE-β-CD towards RLZ compared to α-CD. A solubility growth of 1.7-fold and 3.7-fold with α-CD and SBE-β-CD, respectively, was detected in the solutions of 1% cyclodextrins and accompanied by the permeability reduction. For 1% CD solutions, several biopolymers (1% *w*/*v*) were tested for the membrane permeability under static conditions. The synergistic positive effect of α-CD and polymer on the solubility accompanied by unchanged permeability was revealed in RLZ/α-CD/PG, RLZ/α-CD/PEG400, and RLZ/α-CD/PEG1000 systems. Solid RLZ/CD complexes were prepared. Dynamic dissolution/permeation experiments for the solid samples disclosed the characteristic features of the release processes and permeation rate through different artificial membranes. The maximal permeation rate was determined across the hydrophilic semi-permeable cellulose membrane followed by the lipophilic PermeaPad barrier (model of intestinal and buccal absorption) and polydimethylsiloxane-polycarbonate membrane (simulating transdermal delivery way). Different mode of the permeation between the membranes was estimated and discussed.

## 1. Introduction

Riluzole (2-amino 6 (trifluoromethoxy)benzothiazole) was approved by the Food and Drug Administration in 1995 for the treatment of amyotrophic lateral sclerosis [1]. The effect of riluzole is derived from its ability to block glutamate release and enhance glutamate reuptake leading to the inhibition of glutamate-dependent signaling [2]. The recommended daily dose for adults including aging humans is 100 mg (50 mg every 12 h). With a further increase in dose, the therapeutic effect increases slightly. Riluzole is capable of blocking the voltage-dependent sodium channels in a dose-dependent manner [3]. In addition, the efficacy of riluzole in different types of cancer including the liver, skin, breast, pancreas, colon, bone, brain, lung and nasopharynx has been proven [4]. Riluzole is available on the market in the form of tablets (Rilotec, Riluzole) and also in liquid (suspension) dosage form (Teglutic). The latter is more convenient for patients with impaired swallowing. For the patients with dysphagia the difficulties in swallowing lead to a worsening of therapeutic outcomes. Crushing the riluzole tablet and mixing it with food may have an impact on absorption characteristics, cause dosage errors and the risk of respiratory infections [5]. It may be hazardous, frequently falls outside of the product license for the drug and can have an anesthetic effect on the tongue [6]. Moreover, in the case of crushing the prolonged action of the drug can be violated. A 5 mg/mL oral suspension of riluzole was formulated for patients with dysphagia to make the treatment easier [7].

The partition coefficient of riluzole has been reported from different sources as logP = 2.51 [8] or logP = 3.48 [9], and it is considered as very slightly soluble at pH 6.8 (phosphate buffer) which can be crucial for permeability through the membranes and absorption, thereby limiting application.

To improve the solubility of hydrophobic drugs, various approaches utilizing different auxiliary agents have been reported [10,11,12,13,14,15,16,17,18,19,20]. Among others, cyclodextrins open wide prospects not only of improving the solubility and regulation of drug release but also minimizing gastrointestinal and ocular irritation, and reducing or eliminating unpleasant taste and smell. Depending on the number of glucose residues in the cycle, CDs are classified as α-CD (6 units in the cycle), β-CD (7 ones), and γ-CD (8 ones). As a result of structural changes and rearrangements of a hydrophilic external surface a huge number of cyclodextrin derivatives with different modifications have been synthesized since the early stages of drug delivery [21,22,23,24,25]. Cyclodextrins can function as hosts for polar and nonpolar guests, drug molecules, and polymers [26]. Since the use of cyclodextrins in drug formulations is limited especially for low-dose drugs [27], additions of biopolymers can be advantageous for solubility and dissolution enhancement [28].

The presence of cyclodextrins influences not only the solubility, but also membrane permeability [29]. The importance of evaluating the solubility/permeability trade-off in vitro in the early stages of the drug delivery process is undeniable since it can help to avoid unwanted variations in permeability and allows to cut down the expenses on the costly in vivo experiments. As it was demonstrated for different cyclodextrins [29,30,31], being an advantageous tool in solubility enhancement, in many cases (but not always) cyclodextrins reduce membrane permeability, as a result of decreasing the free (uncomplexed) fraction of the drug molecules [32]. In this case, using the water-soluble biopolymers that interact with CDs, replacing the drug molecules from CDs and increasing the free fraction of drug available for permeation can be useful. The formation of α-CD complexes with both hydrophilic and hydrophobic polymers with small cross-sectional areas (PEG of molecular weight higher than 200, for example) was reported and approved by different techniques [33]. Similarly, the complexation of HP-β-CD with the micellar component of biorelevant media–taurocholate [34] was reported. Using a specific cyclodextrin serves as a fine turning instrument for improving the properties of a specific drug, as it was proved, for example, in our previous study of model drug iproniazid for which the solubility was increased both with hydroxypropyl-β-CD and methylated β-CD, but the permeability was reduced with the first and improved with the second cyclodextrin [35]. As follows, the drug delivery systems based on cyclodextrins and biopolymers are advantageous for controlling the transport properties (solubility, dissolution profile, diffusion rate across the biological membranes) of hydrophobic compounds. The complexes of riluzole with hydroxypropyl-β-cyclodextrin, β-cyclodextrin [36] and 2,6-di-*O*-methyl-β-cyclodextrin [37] were studied and demonstrated the improved water solubility and dissolution rate.

In this study, the complex formation of riluzole (RLZ) with α-cyclodextrin (α-CD) and sulfobutylether-β-cyclodextrin (SBE-β-CD) in solution and in solid state was investigated to improve solubility, dissolution rate and to reveal the effect of complexation on the permeability across the artificial membranes. SBE-β-CD was selected due to its high potential in designing solid oral dosage forms for difficult-to-formulate drugs [38], and as a novel parenterally safe solubilizer and stabilizer [37]. The findings of Rajewski et al. [39,40] showed that sulfoalkylether derivatives gave no observable effects in acute toxicity studies and no negative renal histopathology. In its turn, α-CD being also a recognized and validated pharmaceutical excipient [41] proposed for oral administration [42,43] was taken for the sake of comparison as a representative of CDs composed of 6 α-D-glucopyranose blocks with the less diameter of a hydrophobic cavity among the native CDs. Since the drug/CD association constant cannot provide exhaustive information on the prior factors determining the driving force of the host–guest inclusion phenomenon at a molecular level, in the present study this issue was disclosed via the thermodynamic study of RLZ–SBE-β-CD binding. Thermodynamic aspects of solubilization and complex formation phenomena were derived from the experiments performed at different temperatures. The effect of cyclodextrins on the static state permeation through the hydrophilic cellulose membrane MWCO 12–14 kDa was disclosed. In addition, the three-component systems of RLZ with cyclodextrin and biopolymers of different natures were tested for the effect on the diffusion process in static conditions. Dynamic dissolution/permeation examinations of solid samples of RLZ, RLZ/cyclodextrin physical mixtures and complexes using the cellulose membrane revealed the effect of complex formation on the kinetic parameters. RLZ/cyclodextrin complexes were also subjected to the dissolution/permeation tests with the lipophilic membranes (PermeaPad barrier and polydimethylsiloxane-polycarbonate membrane) closer to the “real” biologic barriers in order to disclose the role of lipophilic membrane properties on the permeation rate of RLZ. Taking into account that to the best of our knowledge the reports devoted to disclosing the permeation of RLZ are rare in the literature, we hope our findings will be helpful for further improving RLZ delivery.

**Figure 1:**
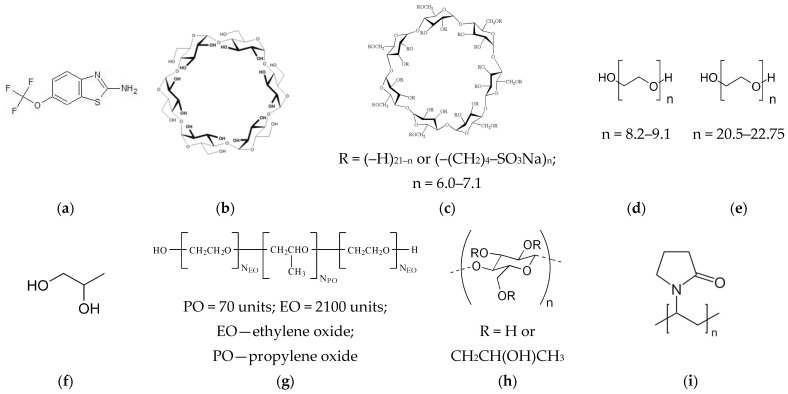
Structures of RLZ, cyclodextrins and polymers used in this study: (**a**) riluzole (RLZ), (**b**) α-cyclodextrin (α-CD), (**c**) sulfobutylether-β-cyclodextrin (SBE-β-CD), (**d**) polyethylene glycol 400 (PEG 400), (**e**) polyethylene glycol 1000 (PEG 1000), (**f**) propylene glycol (PG), (**g**) pluronic F127 (F127), (**h**) hydroxypropylmethylcellulose (HPMC), (**i**) polyvinylpyrrolidone 29–32 (PVP).

The structures of RLZ, cyclodextrins and polymers are illustrated in Figure 1.

## 2. Materials and Methods

### 2.1. Materials

Riluzole (RLZ), (C_8_H_5_F_3_N_2_OS), purity 98%, sulfobutylether-β-CD (SBE-β-CD, purity 99%) were received from BLDpharm (https://www.bld-pharm.com/ (accessed on 2 April 2019), pluronic F127 (F127, M_w_ = 12,600) was purchased from Acros Organics, α-cyclodextrin (α-CD, purity 98%), polyethylene glycol 400 (PEG 400, C_2n_H_4n+2_O_n+1_, n = 8.2–9.1, M_w_ = 380–420), polyethylene glycol 1000 (PEG 1000, C_2n_H_4n+2_O_n+1_, n = 20.5–22.75, M_w_ = 950–1050), polyvinylpyrrolidone K29–32 (PVP, M_w_ = 58,000), propylene glycol (PG, C_3_H_8_O_2_, M_w_ = 76.09), hydroxypropylmethylcellulose (HPMC, M_w_ ~ 10,000) were obtained from Sigma-Aldrich (St. Louis, MO, USA).

Potassium chloride (purity ≥99%), potassium dihydrogen phosphate (purity ≥99%), hydrochloric acid (0.1 mol·dm^−3^), sodium hydroxide (purity ≥98%), sodium acetate (purity ≥99%), and glacial acetic acid (purity ≥99%) were received from Merk. The solvents and reagents were used as received without purification. Buffer preparation procedures were as follows. KH_2_PO_4_ (27.22 g in 1 L)—250 mL and NaOH (2 g in 250 mL)—112 mL were dissolved in 1 L of bidistilled water (2.1 μS cm^−1^ electrical conductivity) for preparing the phosphate buffer pH 6.8. Buffer solution pH 2.0 was made of 6.57 g of KCl dissolved in water with the addition V = 119.0 mL of 0.1 mol∙L^−1^ hydrochloric acid. The volume of the result solution was adjusted to 1 L with water. For the production of the acetic buffer pH 4.0 (I = 0.1 mol·L^−1^) the following procedure was performed. Solution A: 822 mg of sodium acetate was dissolved in 100 mL of water; solution B: 1.44 mL of glacial acetic acid was dissolved in 250 mL of water. Then, 100 mL of solution B was titrated with 20 mL of solution A. An FG2-Kit pH meter (Mettler Toledo, Greifensee, Switzerland) standardized with pH 4.00 and 7.00 solutions was used to check the pH of the prepared buffers.

### 2.2. Determination of RLZ Equilibrium Solubility in CD Solutions

The solubility of RLZ was measured in aqueous buffer solutions pH 2.0, pH 4.0 and pH 6.8 at four temperatures of 298.15 K, 303.15 K, 310.15 K and 313.15 K. To obtain the phase-solubility diagrams according to Higuchi and Connors approach [44] the solutions of 1%, 2%, 3% and 4% of α-CD or SBE-β-CD were prepared and the solubility of RLZ at the equilibrium in these solutions was measured. In addition, the solutions in buffer pH 6.8 containing 1% cyclodextrin and 1% polymer were also taken for the solubility determination. The excess amounts of RLZ were placed into screw-capped vials containing the solutions of a specific content. The vials were continuously shaken in an air thermostat to achieve the equilibrium. After this, the suspensions were settled to avoid supersaturation [45]. The supernatants were centrifuged under temperature control for 20 min at 12,000 rpm (Biofuge pico, Thermo Electron LED GmbH, Langenselbold, Germany), and the concentration of RLZ was determined using a spectrophotometer (Cary-50, Varian, Palo Alto, CA, USA) using the calibration curves with 2–4% accuracy. The experimental results are presented as an average of at least three replicated experiments.

### 2.3. Estimation of RLZ/CD Stability Constant

The stability constants ($K_{C}^{S}$) of the CBZ/CD complex were determined taking into account that the RLZ molecule exists in equilibrium between the free (*C_free_*) and the complexed (*C_complex_*) forms according to the following equilibrium:

*C_total_* = *C_free_* + *C_complex_* (1)

where *C_total_* is the total RLZ concentration, *C_free_* is the free RLZ concentration, *C_complex_* is the concentration of RLZ in the form of the complex with CD. The equilibrium between the free and complexed fractions of RLZ can be expressed by the stability constant, such as:

(2) $$K_{C}^{S}=\frac{C_{complex}}{C_{free}\cdot C_{CD}}$$

where *C_CD_* is the concentration of CD. The stability constant of RLZ/CD complex was derived from the slope of the linear dependence of RLZ equilibrium solubility (*S*_2_(RLZ)) at different concentrations of CD on the CD concentration (*C_CD_*) according to the equation:

(3) $$K_{C}^{S}=\frac{slope}{S_{2}^{0}\cdot \left(1-slope\right)},$$

where $S_{2}^{0}$ is the intrinsic solubility of the drug at a specific pH in pure buffer, and the *slope* is the slope of the phase-solubility diagram. The standard (at 298.15 K) enthalpy change ($\Delta H_{C}^{0,S}$) and the standard entropy change ($\Delta S_{C}^{0,S}$) upon complex formation were calculated with the help of the integral form of the van’t Hoff’s equation:

(4) $$\ln K_{C}^{S}=-\frac{\Delta H_{C}^{0,S}}{RT}+\frac{\Delta S_{C}^{0,S}}{R}$$

where *T* = 298.15 K, and *R* is the universal gas constant. A plot of $\ln K_{C}^{S}$ versus 1/*T* produces $Slope=-\frac{\Delta H_{C}^{0,S}}{R}$ and intercept equal to $\frac{\Delta S_{C}^{0,S}}{R}$. The standard (at 298.15 K) change of the complexation Gibbs energy ($\Delta G_{C}^{0,S}$) was calculated as follows:

(5) $$\Delta G_{C}^{0,S}=-RT\ln K_{C}^{S}$$

### 2.4. Determinations of Thermodynamic Solubilization, Complexation and Solubility Parameters Using a Mole Fraction Scale

In order to implement a proper analysis of both the solubilization and complexation processes in CD solutions from the thermodynamic standpoint, the mole fraction unitary units referring to the standard state were used. The free energy of transferring a compound from the aqueous buffer solution to the CD solution of a certain CD concentration (*C_CD_*) at standard temperature 298.15 K (corresponds to solubilization process) was calculated by Equation (6):

(6) $$\Delta G_{slbz}^{0,X}(C_{CD})=-RT\ln\frac{X_{2}}{X_{2}^{0}},$$

where $X_{2}^{0}$ is the intrinsic solubility of the drug in the absence of CD at 298.15 K and $X_{2}$ is the total solubility in the presence of CD at the same temperature (both expressed in mole fractions), *R* is the universal gas constant. The solubilization enthalpy and entropy were calculated as follows:

(7) $$\Delta H_{slbz}^{0,X}(C_{CD})=\Delta H_{sol}^{0,X}(C_{CD})-\Delta H_{sol}^{0,X}$$

(8) $$T\Delta S_{slbz}^{0,X}(C_{CD})=T\Delta S_{sol}^{0,X}(C_{CD})-T\Delta S_{sol}^{0,X}$$

where $\Delta H_{sol}^{0,X}$ and $\Delta S_{sol}^{0,X}$ are the enthalpy and entropy of solubility process of RLZ in the absence of CD, whereas $\Delta H_{sol}^{0,X}(C_{CD})$ and $\Delta S_{sol}^{0,X}(C_{CD})$ are the enthalpy and entropy of solubility process of RLZ in the presence of CD with concentration *C_CD_*. The solubility thermodynamic parameters were determined from the temperature dependences of the RLZ equilibrium solubility in pure buffer solution using the van’t Hoff equation in the following way:

(9) $$\partial (\ln X_{2}^{0})/\partial T=\Delta H_{sol}^{0,X}/RT^{2}$$

(10) $$\Delta G_{sol}^{0,X}=-RT(\ln X_{2}^{0})$$

(11) $$\Delta G_{sol}^{0,X}=\Delta H_{sol}^{0,X}-T\Delta S_{sol}^{0,X}$$

where $X_{2}^{0}$ is the RLZ molar fraction of the saturated solution in pure buffer, $\Delta H_{sol}^{0,X}$ is the apparent solution enthalpy, $\Delta G_{sol}^{0,X}$ is the apparent solution Gibbs energy at a standard temperature of 298.15 K, *R* is the universal gas constant, $\Delta S_{sol}^{0,X}$ is the apparent solution entropy at 298.15 K. For the complexation process the change of the Gibbs energy in mole fraction scale ($\Delta G_{C}^{0,X}$) was calculated by the following equation:

(12) $$\Delta G_{C}^{0,X}=-RT\ln K_{C}^{X}$$

where $K_{C}^{X}$ is calculated by using the mole fraction concentrations. The $\Delta H_{C}^{0,X}$ and $\Delta S_{C}^{0,X}$ parameters were also derived.

### 2.5. In Vitro Permeation Experiments under Static Conditions

The permeation experiments were carried out at 310.15 K with pH 6.8 of the donor solution. In order to determine the apparent permeability coefficients of RLZ upon the diffusion through the membrane the vertical type Franz diffusion cell (PermeGear, Inc., Hellertown, PA, USA) of 7 mL volume of the donor compartment with 0.785 cm effective surface area was used. RLZ donor solution concentrations were prepared to achieve 43% of the saturation solubility (*C*_0_ = 9.99·10^−4^ M) with and without cyclodextrins and polymers. In particular, the donor solution contained a fixed RLZ concentration in all the experiments. To reveal the impact of CD concentration on the permeability coefficient of RLZ, several concentrations of CDs (0.01 M, 0.02 M, 0.03 M) in the donor solution were applied (two-component systems). For the three-component systems, the donor solutions contained RLZ (*C*_0_ = 9.99·10^−4^ M), 0.01 M CD (α-CD or SBE-β-CD) and 1%*w*/*v* of polymer (6 polymers were tested).

The regenerated cellulose membrane with a molecular weight cut-off (MWCO) 12–14 kDa (Standard Grade RC Dialysis Membrane, Flat Width 45 mm) designated as (RC) was mounted between the donor and receptor chambers. The experiments lasted 5 h; each 30 min the aliquots of 0.5 mL were taken from the receptor chamber and immediately filled with the same volume of the fresh medium. The withdrawn samples of the receptor solutions were subjected to the spectroscopic analysis (UV/VIS spectrophotometer Spectramax 190; Molecular Devices Corporation, San Jose, CA, USA) in 96-well UV black plates (Costar) or by HPLC using the calibration curves. The sink conditions were maintained throughout the experiment meaning that the drug concentration in the acceptor chamber did not exceed 10% of the drug concentration in the donor chamber at any time. A steady state flux (*J*) across the membrane was derived from the linear part of the plot correlating the RLZ amount permeated (*Q*) and time (*t*) taking into account the permeation area (*A*). The permeability coefficient (*P_app_*) was expressed as the steady state flux normalized by the initial concentration of the solution (*C*_0_) such as:

(13) $$P_{app}=\frac{J}{C_{0}}$$

The apparent permeability coefficients were taken as the average values of no less than 3 replicas.

### 2.6. Permeability Calculation Using a Quasi-Equilibrium Transport Model

In order to describe the solubility–permeability tradeoff obtained from the experimental data on the equilibrium solubility and permeability under the static conditions of RLZ with and without cyclodextrins in the solution, a quasi-equilibrium mathematical mass transport model proposed in the studies of Dahan, Miller and co-workers [29,46] for the solubility-enabling formulations containing cyclodextrins was applied. To this end, the apparent membrane permeability ($P_{app}^{calc}$) in the presence of a specific CD concentration was calculated such as:

(14) $$P_{app}^{calc}=\frac{P_{app}^{0}\cdot S_{2}^{0}}{S_{2}}$$

where $S_{2}^{o}$ is the intrinsic solubility of the drug in pure buffer, $S_{2}$ is the solubility of the drug in the presence of CD in the solution.

### 2.7. Preparation of the Solid Samples by Mechanical Grinding Procedure

The equimolar physical mixture of the two components was prepared by mixing parent RLZ with α-CD or SBE-β-CD in a 1:1 ratio using a spatula. The ground samples of RLZ and complexes were processed from parent RLZ and physical mixtures with cyclodextrins. Agate jars (12 mL) with milling balls (agate, 5 mm) and 50–60 mg of the sample were placed in a Planetary ball micro mill (Fritsch Pulverisette 7, Burladingen, Germany) for 1 h with a pause period to avoid the mechanical heating. The rotational speed was 600 rpm. The resulting products were collected and subjected to DSC and PXRD characterization. The samples for the dissolution/permeation examination were additionally sieved through 150 mm mesh to obtain a size distribution of 80–150 mm mesh. The samples were stored in a desiccator with sunlight protection.

### 2.8. Differential Scanning Calorimetry

The DSC profiles of the samples were obtained with the help of a differential scanning calorimeter (Perkin Elmer DSC 4000, Perkin-Elmer Analytical Instruments, Norwalk, CT, USA) with a refrigerated cooling system (USA). The samples were heated in standard aluminum sample holders. The heating rate of 10 K·min^−1^ was applied. The experiments were carried out in a nitrogen atmosphere. The unit was calibrated with indium and zinc standards. The accuracy of the weighing procedure was ±0.01 mg. No less than 3 replicated experiments were carried out for each sample. The average value was taken for the heat of fusion evaluation. The amount of the drug was approximately 0.3 mg.

Based on the DSC data, the relative degree of crystallinity in physical mixtures and ground samples was evaluated by calculating a percent of the ratio between the heat of fusion of RLZ in the sample and that of raw (untreated) RLZ according to the equation proposed in [47]:

(15) $$RLZcrtystallinity(\%)=\frac{\Delta H_{m}(sample)}{\Delta H_{m}(RLZraw)}\cdot 100\%$$

where $\Delta H_{m}(sample)$ and $\Delta H_{m}(RLZraw)$ are the heats of melting for RLZ in test samples and raw (untreated) RLZ, respectively.

### 2.9. PXRD Analysis

The powder XRD data of the bulk materials were recorded under ambient conditions on a D2 Phaser (Bragg-Brentano) diffractometer (Bruker AXS, Karlsruhe, Germany) operating at 30 kV and 10 mA with a copper X-ray source (λCuKα1 = 1.5406 Å) and a high-resolution position-sensitive LYNXEYE XE T detector. The quantity of the sample used for the PXRD analysis was 50 mg. The samples were placed into the poly(methyl metacrylate) plate sample holders and rotated at a speed of 15 rpm during the data acquisition. The data were collected from 4 to 30° 2θ with a step size of 0.02° and a count time of a least 1 s per step.

### 2.10. Dissolution Experiments under Dynamic Conditions

The dynamic evaluation of the dissolution rate from the solid samples containing the amount of RLZ corresponding to a daily dose of 100 mg was carried out in a side-by-side cell (PermeaGear.de H1C SIDE-BI-SIDE Diffusion System, SESGmbH-Analytical system, Bechenheim, Germany). The volume of the donor compartment was 7 mL. The powdered samples were placed in the donor cell. Then, 7 mL of buffer pH 6.8 was added to the donor compartment (start of the experiment). The solution was stirred with a stirring bar at a fixed speed of 500 rpm. The cell was maintained at 310.15 K during the experiment via water circulating. The samples of the solutions (V = 0.4 mL) from the donor cell were withdrawn every 5, 10, 15, 20, 25, 30, 40, 60, 90, 120, and 180 min followed by immediate replacement with fresh medium. The experiment lasted 3 h. The samples from the donor cell were filtered (syringe nylon filter, 13 mm diameter, pore size 0.45 μm). The concentrations of the samples were measured in a 96-well UV black plate (Costar) using a spectrophotometer (Spectramax 190; Molecular devices, Molecular Devices Corporation, San Jose, CA, USA). The kinetic curves of the dissolution/release of pure RLZ and RLZ from the solid samples were obtained.

### 2.11. Permeation Examinations under Dynamic Conditions

Evaluation of the permeation from the solid samples containing the amount of RLZ corresponding to a daily dose of 100 mg was carried out using a side-by-side cell (PermeaGear.de H1C SIDE-BI-SIDE Diffusion System, SESGmbH-Analytical system, Bechenheim, Germany). The membrane of regenerated cellulose MWCO 12–14 kDa (Visking dialysis tubing MWCO 12–14 kDa, Medicell Membranes Ltd., London, UK) was applied as a barrier between the donor and receptor cells with an effective surface area of 1.77 cm^2^. In addition, the PermeaPad barrier (PP) (PHABIOC, Germany, https://www.permeapad.com (accessed on 1 February 2023)) and polydimethylsiloxane-polycarbonate (55% polydimethylsiloxane and 45% polycarbonate, 40 μm in thickness) membrane “Carbosil” (PDS) (PENTAMED, Moscow, Russia, www.penta-med.ru) were used in a series of tests. The volume of the receptor compartment was the same as the donor one—7 mL. The donor and receptor cells were assembled with a membrane between them. The receptor compartment was filled with 7 mL of buffer pH 6.8. In the case of the samples with CD, the receptor solution contained the amount of CD equivalent to that in the solid sample. The solution in the receptor cell was stirred at 500 rpm. The constant temperature of 310.15 K was maintained. The samples of the solutions (V = 0.4 mL) from the receptor chamber were withdrawn every 10, 20, 30, 60, 90, 120, and 180 min and replaced with fresh medium. The concentrations of RLZ in the samples were measured by HPLC. The kinetic permeation curves of pure RLZ and RLZ from the solid samples were plotted. The non-sink conditions implying the changing concentration of RLZ in the donor solution were applied, and the parameter of flux (*J*) was used for the comparison of the permeation behavior of the samples.

### 2.12. Dissolution/Permeation Quantitative Parameters Calculations

As a result of the dissolution/permeation experiments, the kinetic curves of the dissolution/release of raw RLZ and RLZ solid samples were obtained and quantitatively compared through several parameters. The dissolution performance parameter (DPP) [48], was calculated as follows:

(16) $$\mathrm{DPP}=\frac{{\mathrm{AUC}}_{\mathrm{actual}}}{{\mathrm{AUC}}_{\mathrm{theoretical}}}\times 100\%$$

where AUC_actual_ is the integral area under the curve C_0_C_t_, and AUC_theoretical_ is the integral area under the curve C_max_C_max_. Dissolution efficacy after 60 min (DE_60_)—a percentage of the area of the rectangle described by 100% dissolution at 60 min was determined by the calculation of the area under a dissolution curve up to 60 min by the following equation [49]:

(17) $${\mathrm{DE}}_{60}=\frac{\int_{0}^{60}Qdt}{Q_{100\%}\times 60}\times 100\%$$

where *Q* is the percentage of the dissolved drug, *t* is the dissolution time point at 60 min. In addition, a ratio between DE_60_ of the studied systems (DE_60_(test)) and of the reference value for raw RLZ (DE_60_(R)) was calculated for each system as follows:

(18) $${\mathrm{DE}}_{60}\mathrm{ratio}=\frac{{\mathrm{DE}}_{60}\left(\mathrm{test}\right)}{{\mathrm{DE}}_{60}\left(R\right)}$$

In order to simplify a comparison of RLZ dissolution profiles in different formulations a pair-wise procedure was applied to estimate the similarity factor (*f*_2_) proposed by Moore and Flanner [50]:

(19) $$f_{2}=50\times \log\left\{{\left[1+\left(\frac{1}{n}\right)\sum_{j=1}^{n}{\left|R_{j}-T_{j}\right|}^{2}\right]}^{-0.5}\times 100\right\}$$

where *n* is the sampling number, *R* and *T* are the % dissolved of the reference (RLZ_raw in this study) and test products at each time point *j*.

Quantitative analysis of the permeation was evaluated using the flux (*J*) through the membrane which was determined as a slope of the permeation profiles representing the amount of permeated compound over the surface area (*dQ*/*A*) versus the time (*t*) in accordance with the equation:

(20) $$J=\frac{dQ}{A\times dt}$$

### 2.13. HPLC Analysis

The samples of the solutions were analyzed by HPLC using the Shimadzu Prominence model LC-20 AD equipped with a PDA detector and a C-18 column Luna^®^ (150 mm × 4.6 mm i.d., 5 μm particle size and 100 Å pore size). The column temperature was 40 °C. Eluent acetonitrile:water at a ratio of 40:60 *v*/*v* was used. An isocratic regime at a flow rate of 1 mL·min^−1^ was applied. The injection volume was 20 μL. RLZ was detected (UV) at 263 nm with a retention time of 4.95 min.

## 3. Results

### 3.1. Solubility of RLZ in Cyclodextrin Solutions, Thermodynamic Considerations

**Figure 2:**
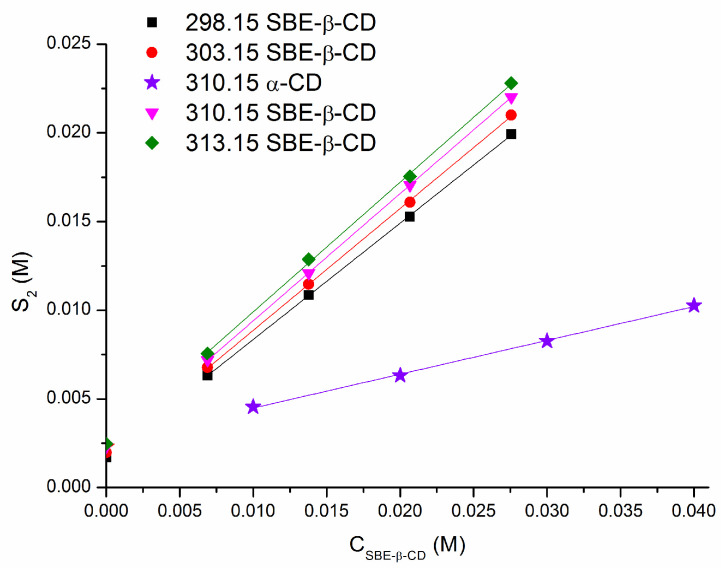
Phase solubility diagrams for RLZ in SBE-β-CD and α-CD solutions at different temperatures in buffer pH 6.8.

The solubility of RLZ in the solutions of pH 2.0 (final pH 2.7), pH 4.0, pH 6.8 and with the additions of α-CD and SBE-β-CD was determined at 310.15 K. In the medium of pH 6.8, the experiments were additionally carried out at 298.15 K, 303.15 K and 313.15 K in pure buffer and in the presence of several SBE-β-CD concentrations in order to disclose the thermodynamic aspects and to gain further knowledge on the mechanism of the inclusion process. The results are listed in Table 1. The phase diagrams in α-CD at 310.15 K and SBE-β-CD at different temperatures in buffer pH 6.8 are illustrated in Figure 2.

The solubility of RLZ (a weak base) was estimated to be pH-dependent in accordance with the ionization constant pK_a_ = 3.8 [9] as follows: pH 2.0 (24.20 mM) > pH 4.0 (6.85 mM) > pH 6.8 (2.31 mM) at 310.15 K. The solubility measured in this study at 298.15 K in buffer pH 6.8 (1.71 mM) is in agreement with the value of 1.61 mM in water reported in the literature [36]. Table 1 demonstrates the RLZ solubility increase upon the growth of the CD concentration and temperature. Figure 2 shows the A_L_ type diagrams indicating the 1:1 inclusion complexes of RLZ with both CDs in a pH 6.8 medium. The phase diagrams obtained at 310.15 K in buffer solutions of pH 2.0 and pH 4.0 for both CDs are provided in the SI file as Figure S1. Notably, the pH of the buffer solution appeared to be a crucial factor in determining the trends of the solubility in the presence of cyclodextrins. Obviously, this comes from the variations of the ionization state of the compound in different pH of the buffers. According to the pK_a_ of RLZ, the particles are fully protonated at pH 2.0, approximately half protonated at pH 4.0 and exist as neutral (uncharged) molecules at pH 6.8. The classical A_L_ type diagram in an acidic medium was obtained only for the 1:1 inclusion complex of RLZ with α-CD in buffer pH 4.0. For the RLZ/α-CD system in buffer pH 2.0, a somewhat A_N_ type diagram is difficult to interpret and, as it was reported by Brewster and Loftsson [51], may be associated with cyclodextrin-induced changes in the dielectric constant of the aqueous complexation media, changes in complex solubility or self-association of cyclodextrin molecules. Specific phase-solubility profiles were derived for the RLZ/SBE-β-CD system in both pH 2.0 and pH 4.0 (Figure S1). A visible deviation of the intercept of the phase solubility diagram from the solubility of RLZ in pure buffers ($S_{2}^{0}$) was obtained (the intercept appeared to fall below the value of $S_{2}^{0}$). Such a case was reported by Loftsson et al. [20] and designated as a non-linear A_L_-type phase-solubility profile with a negative deviation at low cyclodextrin concentrations—$A_{L}^{-}$. The treatment of a diagram by linear regression analysis leads to a negative intercept which is impossible and does not allow for the calculation of the stability constant. Possible reasons for a negative deviation were proposed by Loftsson et al. [52] as self-association of the drug molecules and drug/cyclodextrin complexes, as well as non-inclusion complexation. In conclusion from the results of the phase solubility, we can assert that the simultaneous action of the ionization state of the compound, the complexation ability of cyclodextrin, and the nature and specific features of cyclodextrin (for example, the charge on SBE-β-CD) determines the solubility and phase solubility profile.

The stability constants of RLZ/CD 1:1 inclusion complexes were determined from all the phase solubility diagrams in buffer pH 6.8 and in buffer pH 4.0 for RLZ/α-CD. The results are listed in Table 2.

The value of the stability constant is considered a crucial factor for the absorption of drugs administered in the content of cyclodextrin complexes. Moreover, increasing the cyclodextrin concentration may worsen the bioavailability of the formulation and retard its progression to the market [30]. For poor soluble lipophilic drugs (RLZ belongs to) a moderate binding constant of drug/CD $K_{C}^{S}$ ˂ 5000 M^−1^ [53] is appropriate for successful oral delivery. This is the case of RLZ/SBE-β-CD. At the same time, the drug is rapidly released from the complex with a low binding constant $K_{C}^{S}$ ˂ 200 M^−1^ (RLZ/α-CD in our study) which would reduce bioavailability. Drug/CD complexes with high stability are characterized by a slow rate of drug release leading to a decrease in the free drug fraction available for permeation through the membranes and low absorption.

As follows from Table 2, an 11-fold more stable complex with SBE-β-CD as compared to α-CD was revealed possibly due to a more suitable larger hydrophobic cavity of SBE-β-CD. Interestingly, the comparison with the literature data on the RLZ complexes with HP-β-CD [36], β-cyclodextrin and 2,6-di-*O*-methyl-β-cyclodextrin (DM-β-CD) [37] revealed the following apparent sequence of cyclodextrins according to the complexation ability (stability constants) towards RLZ: HP-β-CD (2327 M^−1^) > DM-β-CD (1609.1 M^−1^) > SBE-β-CD (1116.0 M^−1^) > β-CD (663.2 M^−1^) > α-CD (102.3 M^−1^, 310.15 K). Notably, for all cyclodextrins the experimental temperature was 298.15 K except for α-CD (310.15 K). Besides this, the stability constants borrowed from the literature were obtained in water with an undefined pH that can be meaningful for RLZ as an ionizable substance. As follows, the matter of the observed regularity can be considered qualitative rather than quantitative but allows us to make some informative conclusions. Since the complexation constant for β-CD is essentially greater than that of α-CD, the size of the hydrophobic cavity of the first is more suitable to include the RLZ molecule. The greater value of the association constant with SBE-β-CD (and also HP-β-CD and DM-β-CD) as compared to β-CD makes evident that derivatization of natural CD produces more stable complexes [54]. Concerning SBE-β-CD, it has been recently reported [55] that the charged sulfonate groups of SBE-β-CD molecules are likely to repel one another by extending out and away from each other, providing an additional hydrophobic region near the cavity composed of only the alkyl ether moieties of the sulfobutyl groups. The complexation of neutral molecules (RLZ at pH 6.8 in this study) to this cyclodextrin may occur not only via the cyclodextrin cavity, but also through the alkyl chains near the cavity [56].

The driving forces of the RLZ complex formation were disclosed in the example of SBE-β-CD possessing a more affinity towards RLZ at pH 6.8. To this end, the temperature dependence of ln $K_{C}^{S}$ was plotted (Figure S3) and the thermodynamic parameters of complex formation were calculated by Equations (4) and (5). The following values were derived: $\Delta G_{C}^{0,S}$ = −17.4 (±0.3) kJ·mol^−1^, $\Delta H_{C}^{0,S}$ = −0.7 (±0.0) kJ·mol^−1^, $298.15\cdot \Delta S_{C}^{0,S}$ = 16.7 (±2.2) (kJ·mol^−1^). The stability of the RLZ/SBE-β-CD complex decreases with the temperature growth resulting in the negative enthalpy term and demonstrating an exothermic process. Analysis of the results showed that, in fact, the formation of the complex is completely determined and driven by the entropy: both $\Delta H_{C}^{0,S}$ and $\Delta S_{C}^{0,S}$ are favorable but |$T\Delta S_{C}^{0,S}$| >> |$\Delta H_{C}^{0,S}$| indicating a predominant role of system disordering. Perlovich et al. [57] attributed such a case to a ‘nonclassical’ model of hydrophobic interaction when the impact of reorganization of the solvation shells and the effect of hydrophobic forces upon the complex formation is a crucial factor.

**Figure 3:**
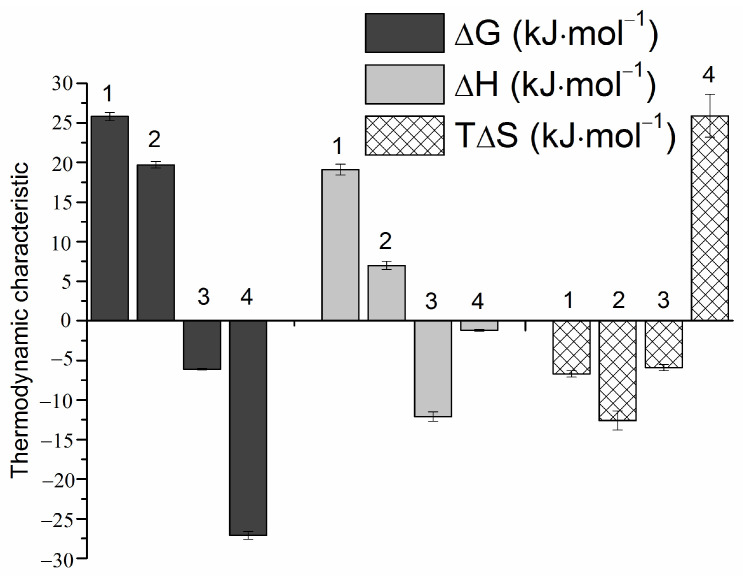
Thermodynamic parameters of RLZ dissolution in pure buffer pH 6.8 (1), dissolution in 4% SBE-β-CD solution at pH 6.8 (2), solubilization in 4% SBE-β-CD solution at pH 6.8 (3), and complexation with SBE-β-CD at pH 6.8 (4) at 298.15 K.

In the next step, the comparative analysis of the dissolution, complexation and solubilization thermodynamics was accomplished similarly to our previous studies [58,59]. To this end, the respective thermodynamic characteristics were calculated by Equations (6)–(12) using the mole fraction unitary units providing thermodynamic quantities for the standard state for RLZ in buffer pH 6.8 and taking the maximal SBE-β-CD concentration (4%) (Table 3). To observe visually the contributions to the driving force of the considered processes, a diagram in Figure 3 was constructed.

As follows from Table 3 and Figure 3, the solubility and dissolution processes are unfavorable ($\Delta G^{0,X}$ > 0) as a result of both unfavorable positive $\Delta H^{0,X}$ and negative $T\Delta S^{0,X}$. As opposed, both solubilization and complexation processes are favorable according to the negative values of $\Delta G_{slbz}^{0,X}$ and $\Delta G_{C}^{0,X}$. At that, since |$\Delta G_{slbz}^{0,X}$| ˂ |$\Delta G_{C}^{0,X}$|, the driving force of complexation is greater indicating the complexation rather than solubilization as a determinative factor of RLZ solubility improvement with SBE-β-CD. Analysis of the enthalpy and entropy contributions revealed that the driving force of the solubilization process is $\Delta H_{slbz}^{0,X}$ = −12.1 kJ·mol^−1^ which is greater than unfavorable negative $T\Delta S_{slbz}^{0,X}$= −5.9 kJ·mol^−1^ (enthalpy driven and determined process). As opposed, the complexation is obviously driven by a great favorable positive entropy ($T\Delta S_{C}^{0,X}$ = 25.9 kJ·mol^−1^), in so doing, the enthalpy takes on an also favorable but rather smaller value ($\Delta H_{C}^{0,X}$ = −1.2 kJ·mol^−1^) providing 22-fold difference between these terms.

### 3.2. Permeability of RLZ in Static Conditions

Permeation coefficients of RLZ were measured in the vertical type of Franz diffusion cell through the cellulose membrane (RC). The same concentration of the drug equal to 9.99∙10^−4^ M was used in the donor solution regardless of the additives of cyclodextrin or both cyclodextrin and polymer in the system. The permeability coefficients were obtained for pure RLZ, in the presence of 0.01 M, 0.02 M, 0.03 M concentrations of α-CD and 0.01 M, 0.02 M of SBE-β-CD, as well as in the systems of 0.01 M cyclodextrin and 1% polymer in buffer pH 6.8. The following biopolymers were used: polyethylene glycol 400 (PEG 400), polyethylene glycol 1000 (PEG 1000), propylene glycol (PG), hydroxypropylmethylcellulose (HPMC), polyvinylpyrrolidone 29–32 (PVP). The polymers were tested to disclose their potential as additional excipients capable of improving the solubility without sacrificing permeability [34,60,61].

The solubility of RLZ in the solutions with the same concentrations of CDs and polymers was also measured in order to disclose the solubility–permeability tradeoff. The results are listed in Table 4.

Analysis of the data in Table 4 demonstrated the reduction in the permeability coefficients upon the increase in CD concentration in the solution. This reduction is more pronounced with SBE-β-CD (3.7-fold for 0.02 M CD) as compared to α-CD (1.5-fold for 0.02 M CD) in full agreement with the increase in the solubility with these cyclodextrins. As it was reported [30], the total permeability across the biological barriers contains three terms: the permeability through the lipophilic membrane per se, and through the adjacent to the membrane unstirred water layers (UWL). In the in vitro conditions of our experiments, the constant stirring of the donor solution at 500 rpm (stirring speed > 50 rpm was shown to be acceptable [62]) was applied so that this layer does not limit the overall permeation. The impact of UWL on the acceptor side can also be considered negligible since the aliquots of the solution from the acceptor compartment were withdrawn every 30 min and replaced with the fresh medium. In this situation, the total permeability is equal to the permeability through the membrane. Cyclodextrins increase the permeation of the lipophilic drugs through the UWL but not through the membrane [63]. This fact explains the reduction in the RLZ permeability in the presence of both cyclodextrins.

Additions of polymers to the 0.01 M CDs solutions resulted in both the solubility and permeability variations. For the solutions with α-CD (0.01 M), the solubility remained unchanged with F127, very slightly reduced with PVP or HPMC, but increased in the presence of: PG (2.25-fold) > PEG400 (1.68-fold) > PEG1000 (1.35-fold). In so doing, the solubility in the α-CD (0.01 M)/PG system was shown to be even greater than in 0.03 M α-CD. Bearing in mind that a high content of CDs in drug formulations should be avoided, this system seems to be a perspective. The permeation experiments from the same solutions demonstrated the unchanged (within the experimental error) permeability coefficients in the presence of PEG400, PEG1000, PG, and HPMC, and reduced ones with F127 (2.05-fold) and PVP (1.16-fold). In the systems with SBE-β-CD (0.01 M) and polymers, the pronounced solubility improvement was not observed—the maximal of 1.14-fold (PEG1000) followed by 1.12-fold (PEG400) is essentially less than in 0.03 M SBE-β-CD without polymers (2.5-fold). At that, in SBE-β-CD (0.01 M) *P_app_* remained the same only with PVP and HPMC but was diminished in the presence of F127 (1.26-fold), PG (1.27-fold), PEG400 (1.41-fold), PEG1000 (1.94). From the above information, it can be concluded that the effect of polymers on the RLZ/α-CD system is more visible as compared to the RLZ/SBE-β-CD one. The solubility increase (occurring even in the presence of small amounts of polymers) is due to the polymer-drug binding and is a result of the formation of water-soluble drug–polymer associates (complexes) [64]. Concerning permeability, the most frequent situation [65] is the simultaneous solubility increase and the permeability decrease. In this study we obtained three systems RLZ/α-CD/PG, RLZ/α-CD/PEG400, and RLZ/α-CD/PEG1000 in which the synergistic positive effect of α-CD and polymer on the solubility was revealed, at that, the permeability was not changed.

This effect can be attributed to the interactions of α-CD and polymer. For example, as shown by Harada [33], α-CD forms complexes, for example, with poly(ethylene glycol) of molecular weight higher than 200 having a large cross-sectional area that might correlate with the cavities of cyclodextrin. Since the RLZ donor solution concentration was constant and was composed of free drug, the drug in the CD complex and the drug associated with polymer, α-CD complexation with PEG, most probably, led to the increasing amount of free RLZ available to diffuse through the membrane that is advantageous for permeation. It can be concluded that just the competitive interactions of polymer and drug with α-cyclodextrin are responsible for the unchanged permeability coefficient upon the increase in solubility in the three-component systems with α-CD and polymers (PG, PEG 400, and PEG 1000).

In the next step, we examined the solubility–permeability interrelation through a quasi-equilibrium mathematical mass transport model developed by Dahan, Miller and co-workers [66] for the solubility-enabling formulations containing different excipients. According to this approach, membrane permeability coefficients ($P_{app}^{calc}$) of RLZ in the presence of CD along and also CD and polymer were calculated by Equation (14). The results are listed in Table 4. The results clearly demonstrated that in the systems with α-CD the calculated values of $P_{app}^{calc}$ are lower than the experimental ($P_{app}$) except RLZ/α-CD/F127 system in which $P_{app}^{calc}$ > $P_{app}$ (1.4-fold) thus allowing to propose a specific mode of interactions between RLZ, α-CD and F127 in this three-component system. Interestingly, the most pronounced differences between the experimental and calculated values with α-CD were estimated for PG (3.1-fold) > PEG400 (2.5-fold) > PEG1000 (1.9-fold), evidently, in accordance with the solubility values. In these cases the impact of solubility on the permeability is inessential.

The solubility/permeation interplay was initially proposed for the two-component solubility-enabling formulations. Equation (14) was taken in our work in order to check its validity on the examples of the RLZ systems with cyclodextrins and polymers in static permeation experiments. Evidently, this equation does not work in the case of three-component systems where the interaction between the solubilizing agents (α-CD with PG and PEGs) takes place. In their turn, in the case of the systems with SBE-β-CD, the calculated values of the permeability coefficients are close to the experimental ones demonstrating a clear interrelation between the solubility increase and permeability decrease proposed by Dahan, Miller and co-workers [66].

### 3.3. Preparation and Characterization of RLZ Solid Samples

**Figure 4:**
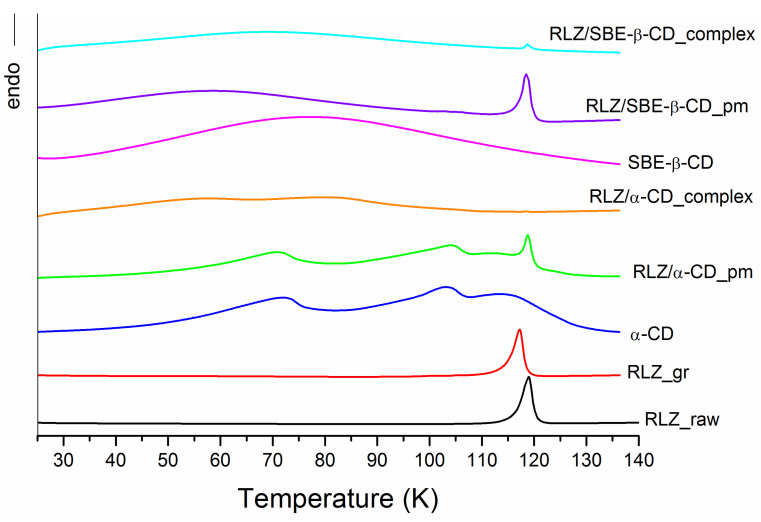
DSC curves for the studied samples: RLZ raw untreated (black), RLZ ground (red), α-CD (blue), RLZ/α-CD physical mixture (green), RLZ/α-CD complex (orange), SBE-β-CD (magenta), RLZ/SBE-β-CD physical mixture (violet), RLZ/SBE-β-CD complex (cyan).

The following solid samples on the basis of RLZ were prepared, characterized by the DSC and PXRD tests and used for dissolution/permeation experimentations: RLZ raw untreated (RLZ_raw), RLZ ground (RLZ_gr), RLZ/α-CD physical mixture (RLZ/α-CD_pm), RLZ/α-CD complex (RLZ/α-CD_complex), RLZ/SBE-β-CD physical mixture (RLZ/SBE-β-CD_pm), RLZ/SBE-β-CD complex (RLZ/α-CD_complex). The DSC curves (in the melting range of RLZ and dehydration of CD) and PXRD of all the samples including both cyclodextrins are provided in Figure 4 and Figure S5 (SI file).

**Figure 5:**
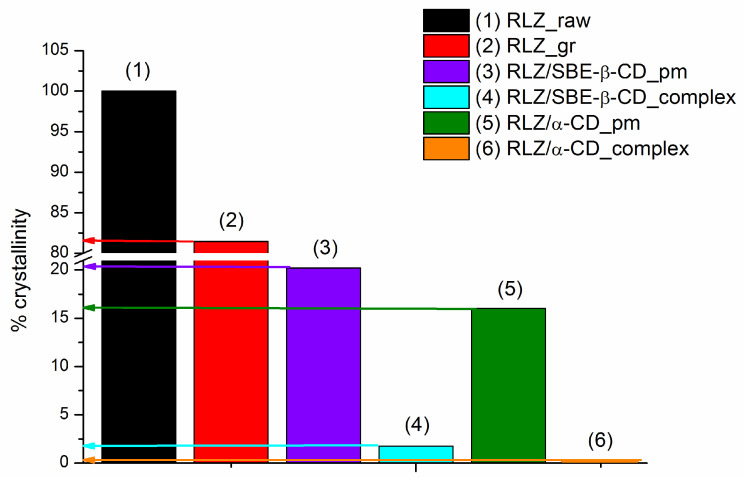
Effect of treatment on the crystallinity of RLZ: (1) RLZ raw untreated (black), (2) RLZ ground (red), (3) RLZ/SBE-β-CD physical mixture (violet), (4) RLZ/SBE-β-CD complex (cyan), (5) RLZ/α-CD physical mixture (green), (6) RLZ/α-CD complex (orange).

Figure 4 demonstrated a sharp endothermic peak between 118 °C and 119 °C typical of a crystalline anhydrous substance corresponding to the melting point of RLZ that is consistent with the literature [36,37]. The PXRD pattern of RLZ (Figure S5) also approved the crystallinity since the high-intensity sharp peaks were detected at 8.95, 13.46, 17.98, 19.22, 20.98, 22.52, 24.97, 26.43 (2Theta) diffraction angles. Expectedly, the ground sample of RLZ partly lost its crystallinity which is evident from both the DSC and PXRD illustrations. In order to assess quantitatively the crystallinity of the samples we determined the melting enthalpies from the DSC curves (discussed below). The thermogram of α-CD provided the endothermic peaks over broad temperature ranges at approximately 48–90 °C and 96–110 °C representing the evaporation of water on the surface and in the interstices as it was also reported by Ho et al. [67]. According to the DSC curve, SBE-β-CD has a characteristic broad endothermal effect of water loss in the range of 70–120 °C similar to that in the study of Ribero et al. [68]. PXRD examination approved the DSC results. PXRD pattern of α-cyclodextrin demonstrated strong crystallization according to the presence of many sharp peaks in the diffractogram [69,70], while the amorphous state of SBE-β-CD is reaffirmed by a hollow-like pattern [68]. The comparative analysis of all the investigated samples of RLZ with raw RLZ has led to the following conclusions. The endothermic melting peak of raw RLZ is slightly shifted by 2 °C towards lower temperatures after grinding accompanied by a slight (20%) crystallinity decrease. As follows from both the DSC (Figure 4) and PXRD (Figure S5) illustrations, the formation of physical mixtures results in essential loss of crystallinity due to the partial complexation at the solid state. This effect became more pronounced for the ground samples with both cyclodextrins. At the same time, the impact of complex formation is greater with α-CD as compared to SBE-β-CD. The effect of sample treatment on the residual share of uncomplexed drug was disclosed using Equation (18), and illustrated in Figure 5.

By inspection of Figure 5, it appears that the presence of cyclodextrins even in physical mixtures leads to RLZ complex formation (more intensive with α-CD, as compared to SBE-β-CD. In the co-ground solid dispersions, practically full complexation with α-CD and slightly incomplete with SBE-β-CD was realized. These results can be attributed to RLZ interactions and complex formation with cyclodextrins in the solid state due to a heating-facilitated decreased action of crystal forces of RLZ dispersed in the cyclodextrin phase. Interestingly, the opposite trends in RLZ-CDs interactions in the solutions and solid state were observed. The complexing and solubilizing ability of SBE-β-CD was shown to be greater than that of α-CD. The value of $K_{C}^{S}$ of RLZ/SBE-β-CD complex was more than an order of magnitude higher than that of RLZ/α-CD (see Table 3). As opposed, the more intensive interaction of α-CD towards RLZ in the solid state was revealed (Figure 5). Our conclusions are consistent with those reported by Mura et al. [47] that the cavity size of cyclodextrin is crucial for drug–CD interactions in solution but can be inessential for the processes in the solid state.

### 3.4. Dynamic Dissolution/Permeation Behavior of RLZ and Its Solid Samples

**Figure 6:**
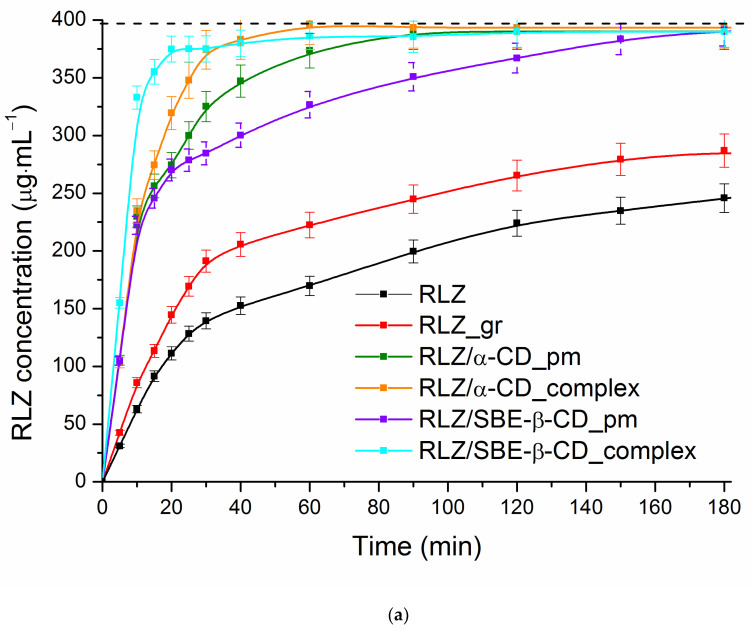

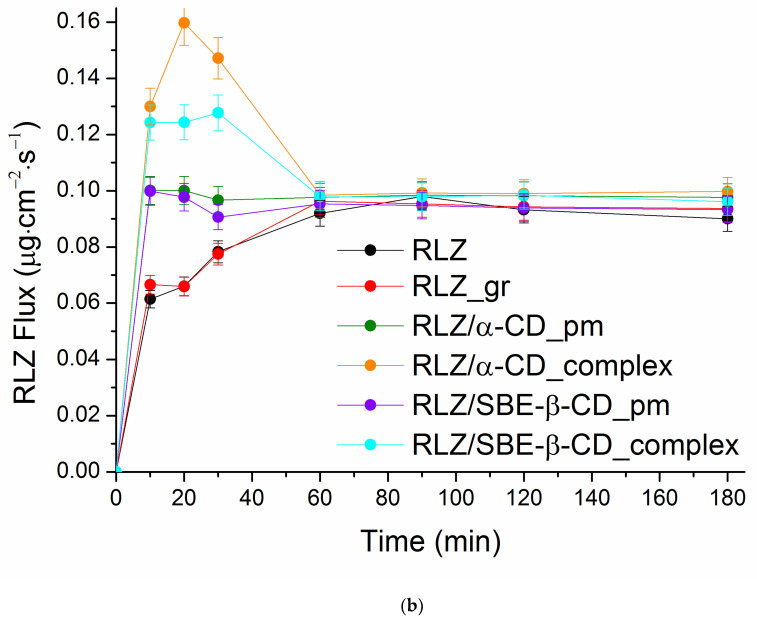
Dissolution curves (**a**) and plots of permeation fluxes (**b**) of RLZ solid samples obtained using the D/P setup at 310.15 K. The RLZ dose concentration is indicated by the dotted line.

The combined dissolution/permeation (D/P) approach has been introduced and tested [71,72,73] as a promising profit-proven tool for the simultaneous evaluation of mutually dependent processes of dissolution and permeation in dynamic conditions. This issue is becoming extremely urgent if cyclodextrins are used in formulations, and the dissolution rather than permeation through the intestinal membrane is often the rate-limiting step in drug absorption [74]. In this study, the dissolution/permeation tests were carried out in buffer solutions pH 6.8 where the solubility of RLZ was shown to be insufficient. First of all, the solid samples of RLZ raw untreated (RLZ_raw), RLZ ground (RLZ_gr), RLZ/α-CD physical mixture (RLZ/α-CD_pm), RLZ/α-CD complex (RLZ/α-CD_complex), RLZ/SBE-β-CD physical mixture (RLZ/SBE-β-CD_pm), RLZ/SBE-β-CD complex (RLZ/SBE-β-CD _complex) were subjected to the experiments in which the cellulose membrane MWCO 12–14 kDa (RC) was used as a barrier. The kinetic curves of the dissolution/release and permeation from the solid samples were obtained and are illustrated in Figure 6a and Figure S5. Figure 6b shows the fluxes derived from the permeation profiles which are informative in terms of better visualization of the variations in the permeation process. Since various formulations of RLZ were studied in this section, to avoid the experimental difficulties associated with quantification of the molecularly dissolved fraction of the drug the flux was used instead of the apparent permeability coefficient usually used to assess the ability to pass the membrane [73].

The experiment lasted 3 h which was enough to trace the kinetic behavior of the samples since in humans the average transit time in the stomach and the small intestine was reported as ca. 12 min and 3–4 h, respectively [75]. Moreover, the preliminary tests demonstrated that according to the solubility of RLZ in buffer pH 6.8 (2.31∙10^−3^ M), it is impossible to dissolve the amount corresponding to the doze of RLZ in 10 mL of pure buffer. At the same time, for the systems of RLZ containing cyclodextrins, three hours were shown to be enough to dissolve the RLZ dose.

To the aim of quantitative evaluation of the dissolution curves, the following parameters characterizing the trends of the dissolution processes were calculated: the amount of RLZ dissolved within 15 min (Q_15_), dissolution efficacy at 60 min (DE_60_), the ratio between DE_60_ of the solid systems and the reference value for raw (untreated) RLZ, the area under the whole dissolution curve (AUC) and dissolution performance parameter (DPP) (Table 5).

As follows from Figure 6a, for raw RLZ and ground RLZ only 62% and 72% of dose, respectively, were dissolved during 180 min. As opposed, all the physical mixtures and complexes demonstrated full dissolution of the dose in this period. According to the amount dissolved within 15 min (Table 5), maximally 88.8% was released from RLZ/SBE-β-CD_complex. Expectedly, 3.13-fold and 13.9-fold smaller amount from raw and ground RLZ samples, respectively, was dissolved within this time. Markedly, only a slight difference between this parameter for both physical mixtures was revealed. From the premises, RLZ/SBE-β-CD_complex can be considered a very rapidly dissolving product (more than 85% released within 15 min), and RLZ/α-CD_complex a rapidly dissolving one [76]. Other solid samples possessed slower release rates. Inspection of Table 5 showed the variations of the DE_60_ parameter, as well as the DE_60_ ratio, from different formulations to follow the Q_15_ trends. Meanwhile, the effective characteristic of the whole dissolution curve–AUC–demonstrated only slightly higher values for the complexes as compared to physical mixtures. The AUC (as well as Q_15_ and DE_60_) of RLZ_gr was slightly increased possibly as a result of decreasing particle size and amorphization upon grinding (see Figure 5). Interestingly as a whole the result of the dissolution/release agreed with the crystallinity variations. Moreover, according to the calculated characterization parameters of the release processes, the release profiles of the physical mixture and complex are closer to each other with α-CD as compared to those with SBE-β-CD. Most probably it can be attributed to the approximately 11-fold lower association constant of RLZ with α-CD, and demonstrates the agreement with the complexation and crystallinity results.

A fixed look at the dissolution (Figure 6a) suggests the existence of several groups of curves according to similarity. This proposal was checked with the f_2_ factor often used for the sake of comparison between the dissolution curves of different samples. We assessed the similarity factor (*f*_2_) by Equation (19) using a pair-wise procedure proposed by Moore and Flanner [50]. As it was stated [77], the values of *f*_2_ > 50 show the similarity of the dissolution profiles. Taking the sample of raw RLZ as a reference, the following consequence of *f*_2_ was derived: 49.9 (RLZ_gr) > 21.9 (RLZ/SBE-β-CD_pm) > 18.7 (RLZ/α-CD_pm) > 15.9 (RLZ/α-CD_complex) > 13.1 (RLZ/SBE-β-CD_complex) demonstrating the similarity of the raw RLZ with RLZ_gr and essential differences with the other samples (the maximal for the complexes). Assuming RLZ/α-CD_pm as a reference, the similarity factor with the rest samples appeared to be 57.4 (RLZ/SBE-β-CD_pm) ≥ 57.1 (RLZ/α-CD_complex) > 40.4 (RLZ/SBE-β-CD_complex). As follows, the systems RLZ/α-CD_pm, RLZ/α-CD_complex and RLZ/SBE-β-CD_pm revealed similar dissolution. In its turn, RLZ/SBE-β-CD_complex kept aloof demonstrating somewhat different from other samples’ dissolution behavior. Analysis of all the calculated parameters allowed us to conclude that both the mechanical effect and the presence of CDs influence the dissolution behavior.

Our experiments on the dissolution/release revealed that the differences between the complexes and physical mixtures are not significant. The issue of the preference for a physical mixture or complex of drugs with cyclodextrin is covered in the literature [78]. It was shown that in physical mixture the complexation can occur during hydration and the formulation may perform well [79]. However, based on their own experience and the results of various literature sources, Miller et al. [78] reported the prevalence of the complex over physical mixture for improved bioavailability in both immediate-release and controlled-release dosage forms. A short comparison of release character between the RLZ complexes from this study and from the literature sources revealed the following: the cumulative amount of drug released from RLZ/α-CD and RLZ/SBE-β-CD complexes within 20 min was lower by 7% and higher by 7%, respectively, than that from β-CD complex [37]; within 10 min—lower by 34% and by 9%, respectively, than that from DM-β-CD [37]; and within 10 min—by 21% lower and by 4% higher than for RLZ/HP-β-CD complex [36]. The data for RLZ/α-CD, RLZ/SBE-β-CD, RLZ/β-CD and RLZ/DM-β-CD release are in accordance with the phase solubility and stability constants. The discrepancy takes place in the stability constant for RLZ/HP-β-CD complex which is more than 2-fold greater than that of RLZ/SBE-β-CD but the amount of the dissolved RLZ within 10 min is practically the same. This can be attributed to the different character of the equilibrium solubility and kinetic dissolution processes.

Chaitanya Mannava et al. [80] declared the higher in vitro flux and diffusion of a drug through an artificial semipermeable membrane (for example, cellulose) as a promising factor in view of high in vivo drug permeability. Cellulose membrane (used in this study) was successfully applied to assess the gastrointestinal and absorptive behavior of drugs in nano- and microparticle formulations [81]. Obviously, the study of permeability in static conditions is a simple and efficient tool especially if the fast screening of the permeation rate for a vast array of multicomponent samples is required. The dynamic scenario is preferable in the case of solid samples since it is believed to abstract from the accounting of the amount of molecularly dissolved drug and accurate monitoring of the flux across the membrane. The results of the permeation from the combined dissolution/permeation setup for RLZ solid samples are illustrated as the cumulative concentrations at each time point (Figure S5) and fluxes (Figure 6b). As follows from Figure S5, the permeation plots are divided into two segments according to the slope and depending on the sample preparation: (1) up to 30 min and (2) from 30 to 180 min. For RLZ_raw and RLZ_gr samples (slope 1) ˂ (slope 2), for RLZ/α-CD and RLZ/SBE-β-CD physical mixtures (slope 1) = (slope 2), and for RLZ/α-CD complex and RLZ/SBE-β-CD complex (slope 1) > (slope 2). A comparison of Figure 6a and Figure S5 allowed us to conclude that just an abrupt increase in the RLZ release from the complexes up to 30 min is a reason behind flux rate enhancement (Figure 6b). In their turn, for the raw and ground samples the flux across the membrane was even slightly (1.3-fold for both) increased. The physical mixtures demonstrating imperceptible variations of flux (Figure 6b) occupy an intermediate position. Proceeding from the assumption (underlined above) that high permeation flux in vitro promises better permeation in vivo, the RLZ/CD complexes rather than physical mixtures seem to be better in view of successful absorption. At that, for RLZ/α-CD_complex the highest increase in RLZ flux was documented most likely due to the less crystallinity of this sample as a result of a more intensive interaction of α-CD with RLZ in the solid state complex as it is written above (Section 3.3). Importantly, the trends in flux across the cellulose membrane (Figure 6b) follow the regularities of the sample’s crystallinity (Figure 5). But the flux differences are less visible.

The motivation for further examinations was based on the following discoveries. From the results of the dynamic dissolution/permeation using the RC membrane (given above), RLZ complexes with both CDs demonstrated a similar character of RLZ release. At the same time, the flux through the membrane was increased to more extent with the α-CD complex. The fluxes of the complexes exceeded those of physical mixtures. Besides this, as a result of the solubility-static permeability experiments, only the systems with α-CD and polymers revealed a synergistic positive effect on the solubility without the decrease in permeability.

**Figure 7:**
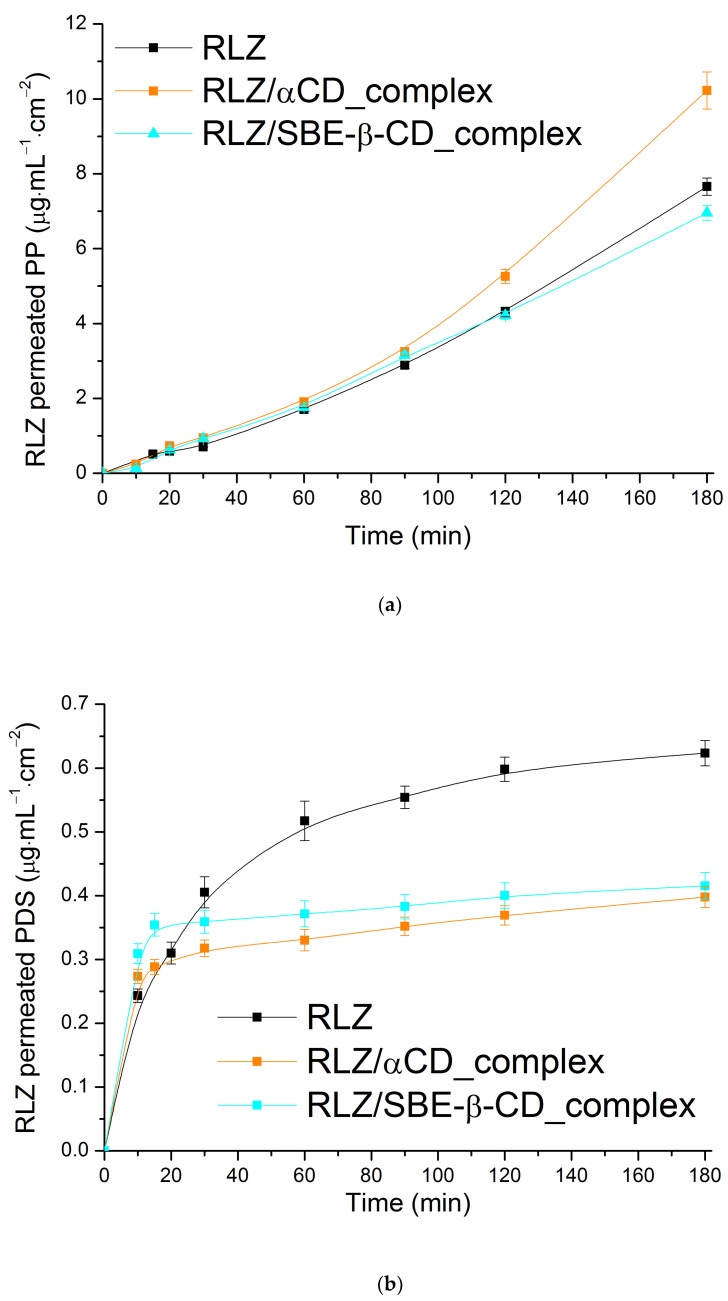
Cumulative amount of RLZ permeated from RLZ_raw, RLZ/α-CD_complex and RLZ/SBE-β-CD_complex: (**a**) PP, (**b**) PDS obtained using the D/P setup at 310.15 K.

Bearing in mind, that the patients taking RLZ may have difficulties swallowing it seems that other delivery apart from the oral path would be applicable in the future and the permeation through the artificial membranes mimicking the intestinal/buccal (PermeaPad barrier) and transdermal (polydimethylsiloxane-polycarbonate) can be of interest. From all the premises, the D/P tests were carried out with the help of lipophilic membranes: PermeaPad barrier (PP) and polydimethylsiloxane membrane (PDS) for RLZ_raw, RLZ/α-CD_complex and RLZ/SBE-β-CD_complex. The results are illustrated in Figure 7.

In order to compare the permeation potential of the membranes used in the experiments the maximal amount of RLZ permeated (Q_max_) was taken and tabulated (Table S1). As expected, the following order of the barriers according to Q_max_ was revealed: RC > PP > PDS. The same regularities were disclosed in our previous study for antidepressants nortryptilin and amitryptilin [82]. The total amount of RLZ permeated from the samples through PP was 38–53% lower in comparison with RC. The same regularity (37–57% difference) was estimated by Wu et al. [83] for caffeine liposomal formulations, but 63–78% for hydrocortisone. The authors proposed the permeation phenomenon with different barriers to be both drug and formulation-dependent. Comparison of Figure S5 and Figure 7a,b clearly demonstrated different modes of RLZ and complexes permeation through the membranes used which allowed us to propose both the membrane nature and possible interactions between the medium solution and membrane components to be crucial for permeation. Consideration of the permeation trends revealed that, as expected, the simplest kind of dependence—almost linear permeation plots (Figure S5)—were obtained with hydrophilic RC membrane as a result of donor solution concentration variations upon the dissolution of an RLZ sample. In this case, the diffusion depends on the concentration gradient between the donor and acceptor compartments of the Franz cell [84]. The plots of the RLZ cumulative amount permeated through the PP barrier from all the samples represented the exponential kinetic dependences close to each other in the time period from zero to 60 min. Next, an abrupt increase took place for RLZ/α-CD_complex (Q_max_ is 1.3-fold greater as compared to RLZ_raw) which is comparable with the similar difference for the RC membrane (Q_max_ is 1.2-fold greater as compared to RLZ_raw). As opposed to the RC membrane, for RLZ/SBE-β-CD_complex with PP barrier, the value of Q_max_ was even slightly reduced in spite of the dramatic growth of the dissolution. Most probably the structure of the membrane lipids in PP suggests that there is a dipole layer between the aqueous phase and the hydrocarbon interior of the membrane (phospholipids and other amphiphilic molecules represent a bilayer in which the polar “head” groups of the lipids appear on the exterior bordering the aqueous phase and the lipophilic “tails” are extended toward the center of the bilayer) which leads to a negative surface potential [84,85]. The negative charges of the membrane surface and SBE-β-CD may retain the drug permeation as a result of charge repulsion. This fact clearly demonstrated the specific features of cyclodextrin used to be also responsible for the permeation mode.

The permeation plots through PDS can be described by the logarithmic function. The permeated amount of RLZ_raw increased gradually, whereas for both complexes the abrupt increase up to 20 min with the following reduction was shown. As a result, a 1.5-fold lower Q_max_ for the complexes as compared to RLZ_raw was estimated (Table S1) in spite of the increased donor concentration. The PDS membrane is known to possess a heterophase and heteropolar structure like human skin epidermis [86]. Most probably, this is the case when the barrier is the limiting step of the permeation, and the synergistic impact of several factors, such as drug–membrane, medium–membrane and drug–CDs interactions influence the permeation.

The results obtained in the present study can improve the prediction of RLZ and related compounds absorption in vivo and provide additional information on possible alternative delivery.

## 4. Conclusions

The aqueous solubility of the anticonvulsant drug riluzole (RLZ) was improved with α-cyclodextrin (α-CD) and sulfobutylether-β-cyclodextrin (SBE-β-CD). A greater affinity of SBE-β-CD as compared to α-CD towards RLZ was revealed. Reduction in the RLZ permeability through cellulose membrane with cyclodextrins in solution under static conditions was estimated. Screening of various biopolymers (1% *w*/*v*) showed an increase in solubility without loss of permeability in RLZ/α-CD/PG, RLZ/α-CD/PEG400, and RLZ/α-CD/PEG1000 three-component systems. In view of the solubility enhancement with cyclodextrins, the complexes RLZ in the solid state were prepared. Dissolution/permeation experiments under dynamic conditions using three kinds of artificial membranes were carried out using a side-by-side Franz diffusion cell. The results of the dissolution/release were characterized by several quantitative parameters and appeared to agree with the variations of the sample’s crystallinity and phase-solubility behavior. RLZ/α-CD_complex demonstrated the highest increase in RLZ flux. Comparative analysis of the diffusion across different membranes testified to the synergistic impact of several factors, such as drug–membrane, medium–membrane and drug–CDs interactions that are responsible for the permeation rate in the case of the barriers with complicated structures. Taking into account that the reports devoted to disclosing the permeation of RLZ are rare in the literature, we hope our findings will be helpful for further improving RLZ delivery.

## Figures and Tables

**Table 1:** Solubility of RLZ in buffer pH 6.8 and with SBE-β-CD or α-CD in solution at different temperatures.

*T* (K)	RLZ Solubility (S_2_∙10^3^ M)				
	SBE-β-CD Concentration (C_SBE-β-CD_∙10^3^ M)				
	pH 6.8				
	0	6.89	13.78	20.67	27.56
298.15	1.71	6.32	10.86	15.27	19.92
303.15	1.98	6.77	11.47	16.09	21.01
310.15	2.31	7.19	12.09	17.06	22.02
313.15	2.47	7.56	12.87	17.54	22.80
	pH 4.0				
310.15	6.85	17.16	28.17	42.13	52.47
	pH 2.0 (final pH 2.7)				
310.15	24.20	16.70	24.26 pH	31.99	40.16
		α-CD concentration (C_α-CD_∙10^3^ M)			
	0	10.00	20.00	30.00	40.00
	pH 6.8				
310.15	2.31	4.55	6.32	8.26	10.26
	pH 4.0				
310.15	6.85	10.61	14.26	18.00	22.28
	pH 2.0 (final pH 2.7)				
310.15	24.20	29.34	32.28	33.94	34.72

**Table 2:** Complexation constants ($K_{C}^{S}$) of RLZ/SBE-β-CD and RLZ/α-CD complexes at different temperatures.

*T* (K)	298.15	303.15	310.15	313.15
	pH 6.8			
RLZ/SBE-β-CD	1116.0 ± 7.6	1110.6 ± 10.4	1103.6 ± 2.2	1100.9 ± 19.1
RLZ/α-CD	-	-	102.3 ± 2.1	-
	pH 4.0			
RLZ/α-CD	-	-	92.3 ± 2.5	-

**Table 3:** Solubility, dissolution, solubilization and complexation thermodynamic parameters of RLZ and RLZ/SBE-β-CD system (pH 6.8, 298.15 K).

**Solubility in Buffer pH 6.8 ^1^**			
$X_{2}^{0}$	$\Delta G_{sol}^{0,X}$ (kJ·mol^−1^)	$\Delta H_{sol}^{0,X}$ (kJ·mol^−1^)	$T\Delta S_{sol}^{0,X}$ (kJ·mol^−1^)
3.07∙10^−5^	25.8 ± 0.5	19.1 ± 0.7	−6.7 ± 0.4
**Dissolution in 4% SBE-β-CD (pH 6.8) ^2^**			
$X_{2}$	$\Delta G_{dis}^{0,X}$ (kJ·mol^−1^)	$\Delta H_{dis}^{0,X}$ (kJ·mol^−1^)	$T\Delta S_{dis}^{0,X}$ (kJ·mol^−1^)
3.60∙10^−4^	19.7 ± 0.4	7.0 ± 0.5	−12.6 ± 1.2
**Solubilization in 4% SBE-β-CD (pH 6.8) ^3^**			
$X_{2}/X_{2}^{0}$	$\Delta G_{slbz}^{0,X}$ (kJ·mol^−1^)	$\Delta H_{slbz}^{0,X}$ (kJ·mol^−1^)	$T\Delta S_{slbz}^{0,X}$ (kJ·mol^−1^)
11.74	−6.1 ± 0.1	−12.1 ± 0.6	−5.9 ± 0.4
**Complexation** **^4^**			
$K_{C}^{X}$	$\Delta G_{C}^{0,X}$ (kJ·mol^−1^)	$\Delta H_{C}^{0,X}$ (kJ·mol^−1^)	$T\Delta S_{C}^{0,X}$ (kJ·mol^−1^)
55,954.9	−27.1 ± 0.5	−1.2 ± 0.1	25.9 ± 2.7

${\;}^{1}\ln X_{2}^{0}=(-2.7\pm 0.3)-(2293\pm 90)/T;r=0.9985;\sigma =2.54\cdot 10^{-4};n=4;$${\;}^{2}\ln X_{2}=(-5.1\pm 0.2)-(844\pm 57)/T;r=0.9954;\sigma =1.04\cdot 10^{-4};n=4;$${\;}^{3}\ln(X_{2}/X_{2}^{0})=(-2.4\pm 0.2)+(1449\pm 73)/T;r=0.9975;\sigma =1.69\cdot 10^{-4};n=4;$${\;}^{4}\ln K_{C}^{X}=(10.4\pm 0.0)+(145\pm 8)/T;r=0.9968;\sigma =2.18\cdot 10^{-6};n=4$.

**Table 4:** Solubility (*S*_2_), experimental (*P_app_*) and calculated ($P_{app}^{calc}$) by a quasi-equilibrium mathematical mass transport model permeability coefficients of RLZ in the systems with CD and CD/polymer (1%) in buffer pH 6.8 at 310.15 K.

System	*S*_2_∙10^3^ (M)	*P_app_*∙10^6^ (cm∙s^−1^)	$P_{app}^{calc}$∙10^6^ (cm∙s^−1^)	*S*_2_∙10^3^ (M)	*P_app_*∙10^6^ (cm∙s^−1^)	$P_{app}^{calc}$∙10^6^ (cm∙s^−1^)
	**α-CD**			**SBE-β-CD**		
Buffer	2.31	11.96 ± 0.17	-	2.31	11.96 ± 0.17	-
CD (0.01 M)	4.55	8.86 ± 0.13	6.07	9.44	3.62 ± 0.08	2.93
CD (0.02 M)	6.32	7.96 ± 0.17	4.37	16.39	3.19 ± 0.13	1.69
CD (0.03 M)	8.26	7.34 ± 0.15	3.44	23.59	n.d. ^1^	1.17
CD (0.01 M)/PEG400	7.65	9.02 ± 0.26	3.61	10.59	2.56 ± 0.04	2.61
CD (0.01 M)/PEG1000	6.12	8.63 ± 0.08	4.51	10.78	1.87 ± 0.06	2.56
CD (0.01 M)/PG	10.26	8.38 ± 0.14	2.69	10.09	2.84 ± 0.04	2.74
CD (0.01 M)/F127	4.50	4.32 ± 0.09	6.14	9.94	2.88 ± 0.14	2.78
CD (0.01 M)/PVP	4.18	7.64 ± 0.10	6.61	9.44	3.77 ± 0.11	2.93
CD (0.01 M)/HPMC	4.17	8.84 ± 0.24	6.63	9.64	3.79 ± 0.05	2.87

^1^ not determined.

**Table 5:** Parameters of the dissolution curves: the amount of RLZ dissolved at 15 min (Q_15_), dissolution efficacy at 60 min (DE_60_), the ratio between DE_60_ of the samples and RLZ raw (reference value), the area under the dissolution curve (AUC) and dissolution performance parameter (DPP), 310 K.

System	Q_15_ (%)	*^a^* DE_60_ (%)	*^b^* DE_60_ ratio	AUC (mg·min^−1^)	*^c^* DPP (%)
RLZ_raw	22.9	30.0	-	33.1	46.2
RLZ_gr	28.3	39.9	1.3	40.9	57.0
RLZ/α-CD_pm	64.1	72.1	2.4	63.8	89.0
RLZ/α-CD_complex	68.6	79.2	2.6	66.2	92.3
RLZ/SBE-β-CD_pm	61.4	64.9	2.2	59.3	82.7
RLZ/SBE-β-CD_complex	88.8	85.1	2.8	66.9	93.3

*^a^* $DE_{60}=\frac{\int_{0}^{60}Qdt}{Q_{100\%}\times 60}\times 100\%$; *^b^* $DE_{60}ratio=\frac{DE_{60}(test)}{DE_{60}(R)}$; *^c^* $DPP=\frac{AUC_{actual}}{AUC_{theoretical}}\times 100\%$.

## Data Availability

The results obtained for all experiments performed are shown in the manuscript and SI; the raw data will be provided upon request.

## Supplementary Materials

The following are available online at https://www.mdpi.com/article/10.3390/pharmaceutics16060757/s1, Figure S1: Phase solubility diagrams for RLZ in α-CD and SBE-β-CD in the buffer solutions of pH 2.0 and pH 4.0 at 310.15 K; Figure S2: Temperature dependences of the dissolution in pure buffer (a), dissolution in 4% SBE-β-CD (b), solubilization in 4% SBE-β-CD (c), and complexation with SBE-β-CD (d) of RLZ (pH 6.8, mole fraction scale); Figure S3: Van’t Hoff plots of the complex formation between RLZ and SBE-β-CD at pH 6.8; Figure S4: PXRD patterns of: RLZ raw untreated (black), RLZ ground (red), α-CD (blue), RLZ/α-CD physical mixture (green), RLZ/α-CD complex (orange), SBE-β-CD (magenta), RLZ/SBE-β-CD physical mixture (violet), RLZ/SBE-β-CD complex (cyan); Figure S5: Permeation profiles (amount of RLZ permeated through the RC membrane) of RLZ solid samples obtained using the D/P setup at 310.15 K; Table S1: The maximal cumulative amount (Q_max_) of RLZ permeated through the membranes at 310.15 K.
