# Supplementary material for: Controlling the Solubility, Release Rate and Permeation of Riluzole with Cyclodextrins
Source: Pharmaceutics. 2024 Jun 3;16(6):757. doi: 10.3390/pharmaceutics16060757 (PMC11206789; doi:10.3390/pharmaceutics16060757)
Supplement: Supplementary file 1 [file pharmaceutics-16-00757-s001.zip › pharmaceutics-3032676-supplementary.pdf]

Controlling the solubility, release rate and permeation of riluzole with cyclodextrins

Tatyana V. Volkova, Olga R. Simonova, German L. Perlovich*

[a]G.A. Krestov Institute of Solution Chemistry RAS, 153045 Ivanovo, Russia.

**Corresponding author:1 Akademicheskaya str., 153045 Ivanovo, Russian Federation,*

E-mail: glp@isc-ras.ru

Table of Contents

Figure S1	Phase solubility diagrams for RLZ in α-CD and SBE-β-CD in the buffer solutions of pH 2.0 and pH 4.0 at 310.15 K.	2
Figure S2	Temperature dependences of the dissolution in pure buffer (a), dissolution in 4% SBE-β-CD (b), solubilization in 4% SBE-β-CD (c), and complexation with SBE-β-CD (d) of RLZ (pH 6.8, mole fraction scale).	3
Figure S3	Van't Hoff plots of the complex formation between RLZ and SBE-β-CD at pH 6.8.	3
Figure S4	PXRD patterns of: RLZ raw untreated (black), RLZ ground (red), α-CD (blue), RLZ/α-CD physical mixture (green), RLZ/α-CD complex (orange), SBE-β-CD (magenta), RLZ/SBE-β-CD physical mixture (violet), RLZ/SBE-β-CD complex (cyan).	4
Figure S5	Permeation profiles (amount of RLZ permeated through the RC membrane) of RLZ solid samples obtained using the D/P setup at 310.15 K.	5
Table S1	The maximal cumulative amount (Q_{max}) of RLZ permeated through the membranes at 310.15 K.	5

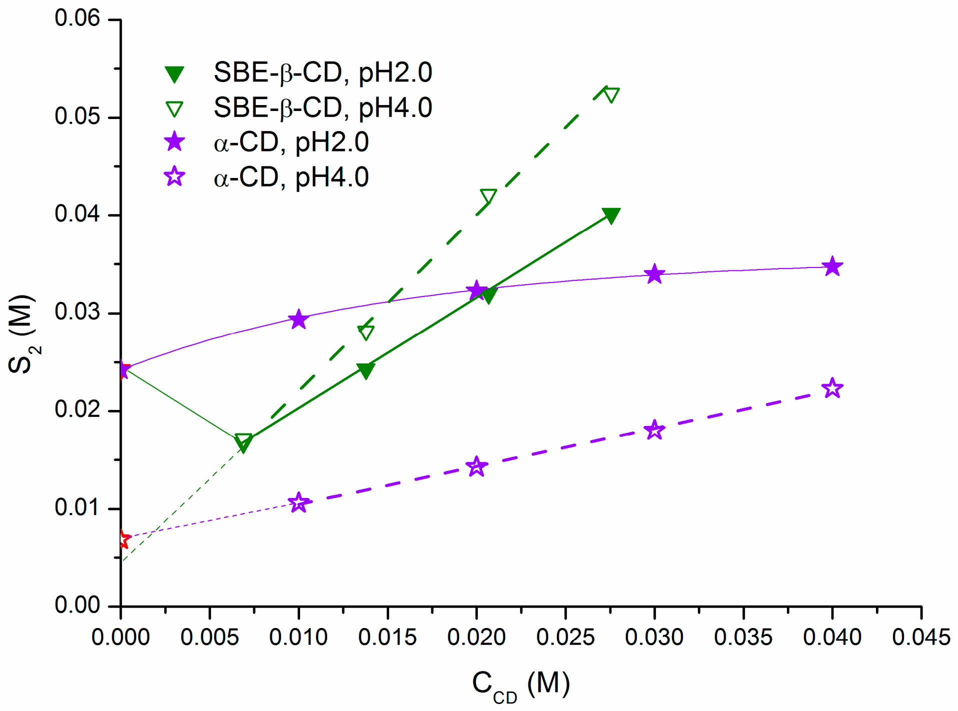

Figure S1. Phase solubility diagrams for RLZ in α-CD and SBE-β-CD in the buffer solutions of pH 2.0 and pH 4.0 at 310.15 K.

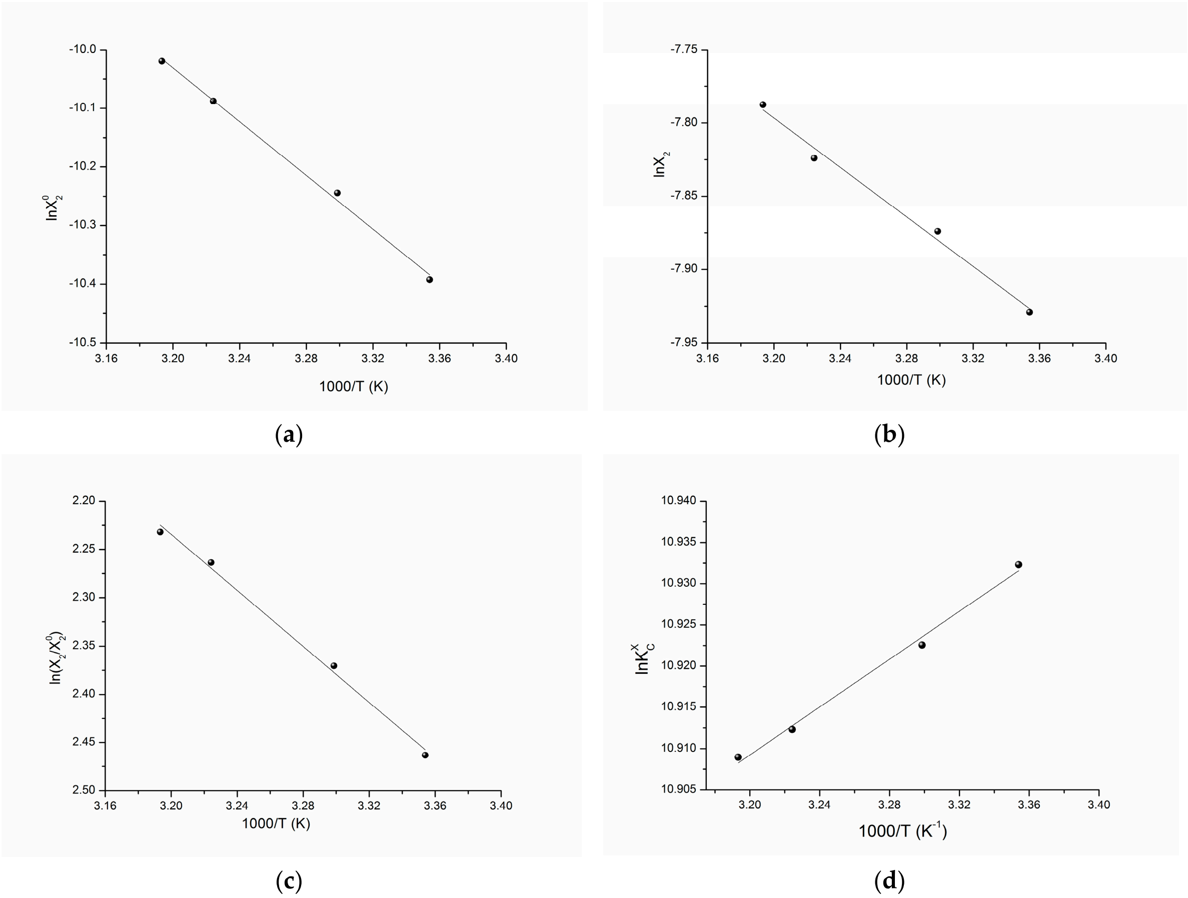

Figure S2. Temperature dependences of the dissolution in pure buffer (a), dissolution in 4% SBE-β-CD (b), solubilization in 4% SBE-β-CD (c), and complexation with SBE-β-CD (d) of RLZ (pH 6.8, mole fraction scale).

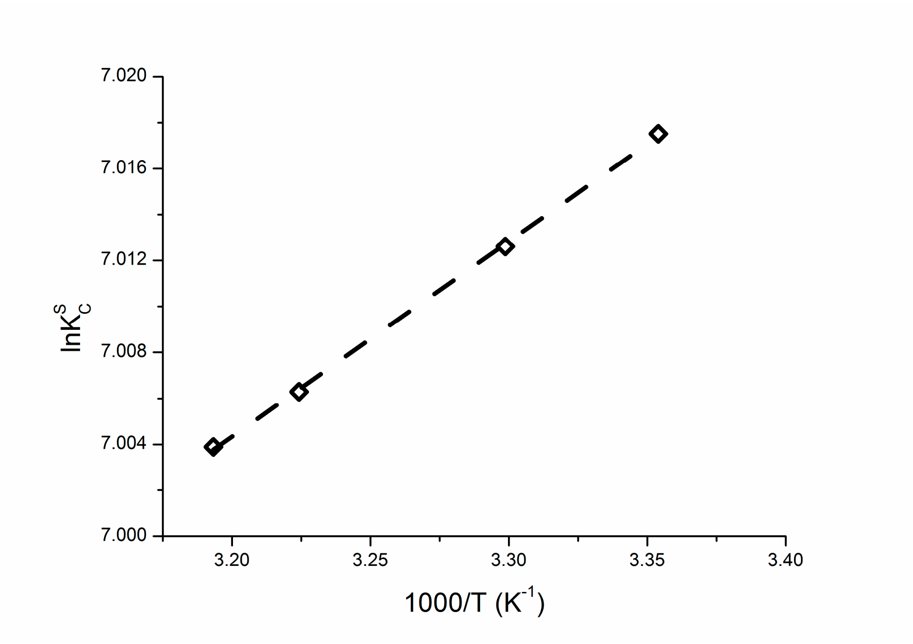

Figure S3. Van't Hoff plots of the complex formation between RLZ and SBE-β-CD at pH 6.8.

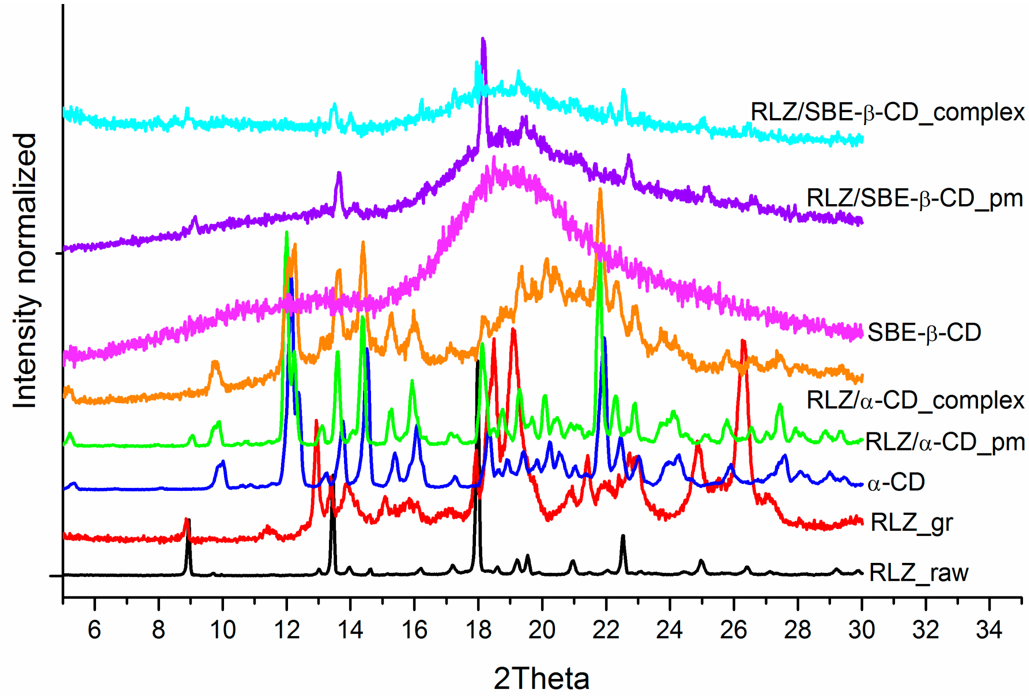

Figure S4. PXRD patterns of: RLZ raw untreated (black), RLZ ground (red), α-CD (blue), RLZ/α-CD physical mixture (green), RLZ/α-CD complex (orange), SBE-β-CD (magenta), RLZ/SBE-β-CD physical mixture (violet), RLZ/SBE-β-CD complex (cyan).

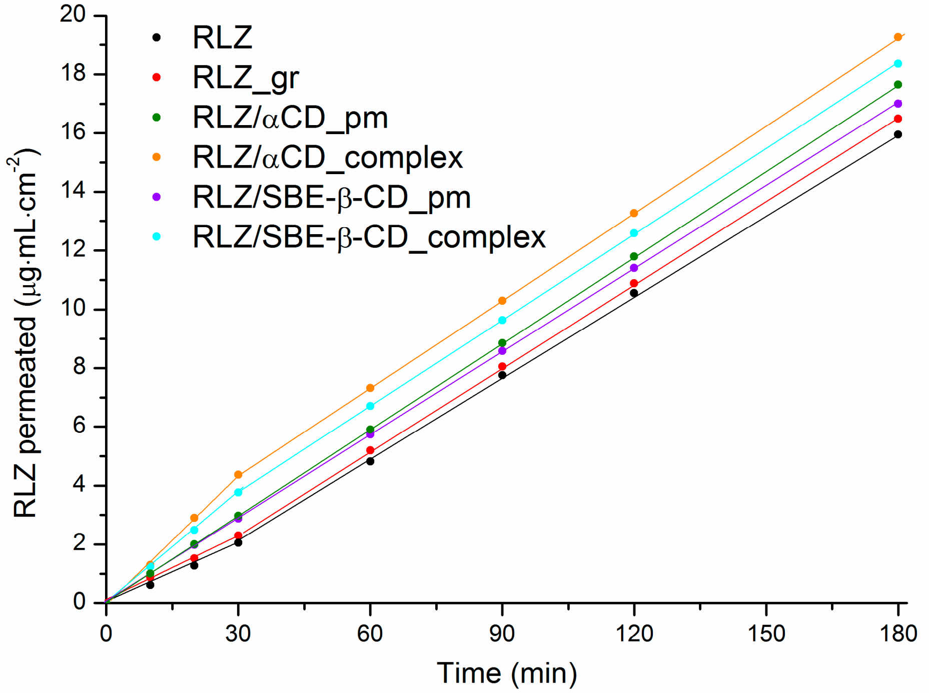

Figure S5. Permeation profiles (amount of RLZ permeated through the RC membrane) of RLZ solid samples obtained using the D/P setup at 310.15 K.

Table S1. The maximal cumulative amount (Q_{max}) of RLZ permeated through the membranes at 310.15 K.

System	Q_{max} ($\mu g \cdot mL^{-1} \cdot cm^{2}$)		
	RC	PP	PDS
RLZ_raw	16.0	7.7	$6.2 \cdot 10^{-1}$
RLZ/α-CD_complex	19.3	10.2	$4.0 \cdot 10^{-1}$
RLZ/SBE-β-CD_complex	18.4	7.0	$4.1 \cdot 10^{-1}$
